# A Multi-Variant, Viral Dynamic Model of Genotype 1 HCV to Assess the *in vivo* Evolution of Protease-Inhibitor Resistant Variants

**DOI:** 10.1371/journal.pcbi.1000745

**Published:** 2010-04-15

**Authors:** Bambang S. Adiwijaya, Eva Herrmann, Brian Hare, Tara Kieffer, Chao Lin, Ann D. Kwong, Varun Garg, John C. R. Randle, Christoph Sarrazin, Stefan Zeuzem, Paul R. Caron

**Affiliations:** 1 Vertex Pharmaceuticals Incorporated, Cambridge, Massachusetts, United States of America; 2 Institute of Biostatistics and Mathematical Modeling, Faculty of Medicine, Johann Wolfgang Goethe University, Frankfurt am Main, Germany; 3 Medizinische Klinik 1, J. W. Goethe-University Hospital, Frankfurt am Main, Germany; University of Oxford, United Kingdom

## Abstract

Variants resistant to compounds specifically targeting HCV are observed in clinical trials. A multi-variant viral dynamic model was developed to quantify the evolution and *in vivo* fitness of variants in subjects dosed with monotherapy of an HCV protease inhibitor, telaprevir. Variant fitness was estimated using a model in which variants were selected by competition for shared limited replication space. Fitness was represented in the absence of telaprevir by different variant production rate constants and in the presence of telaprevir by additional antiviral blockage by telaprevir. Model parameters, including rate constants for viral production, clearance, and effective telaprevir concentration, were estimated from 1) plasma HCV RNA levels of subjects before, during, and after dosing, 2) post-dosing prevalence of plasma variants from subjects, and 3) sensitivity of variants to telaprevir in the HCV replicon. The model provided a good fit to plasma HCV RNA levels observed both during and after telaprevir dosing, as well as to variant prevalence observed after telaprevir dosing. After an initial sharp decline in HCV RNA levels during dosing with telaprevir, HCV RNA levels increased in some subjects. The model predicted this increase to be caused by pre-existing variants with sufficient fitness to expand once available replication space increased due to rapid clearance of wild-type (WT) virus. The average replicative fitness estimates in the absence of telaprevir ranged from 1% to 68% of WT fitness. Compared to the relative fitness method, the *in vivo* estimates from the viral dynamic model corresponded more closely to *in vitro* replicon data, as well as to qualitative behaviors observed in both on-dosing and long-term post-dosing clinical data. The modeling fitness estimates were robust in sensitivity analyses in which the restoration dynamics of replication space and assumptions of HCV mutation rates were varied.

## Author Summary

Hepatitis C virus (HCV) infects an estimated 170 million people worldwide. Current treatment for HCV is 48 weeks of peginterferon and ribavirin of which patient response has large variability. Recently, specifically targeted antiviral therapies for HCV (STAT-C) are under clinical development and have shown potentials to improve response. Within a patient, HCV exists as quasispecies consisting of multiple variants. Models of HCV dynamics in response to peginterferon and ribavirin treatment have been proposed elsewhere, with HCV quasispecies assumed to respond homogenously to treatment. However, some of the HCV variants possess different degrees of sensitivity to a STAT-C compound, and therefore, selections and competitions among variants have been observed in patients treated with STAT-C. We have developed a viral dynamic model that quantifies the evolution of multiple variants in patients dosed in monotherapy with telaprevir, a compound specifically designed to inhibit HCV NS3.4A protease. Our novel modeling approach integrated data from both *in vitro* and in patients, both during and after dosing with telaprevir. Our model quantified the antiviral response to telaprevir and the *in vivo* fitness of variants. The model provides a useful framework for the designs of STAT-C during clinical development and for understanding the consequences of failure to STAT-C.

## Introduction

Hepatitis C virus (HCV) is estimated to infect 170 million people worldwide [1]. Current HCV treatment with pegylated interferon (Peg-IFN) and ribavirin (RBV) for the most common genotype 1 strain requires 48 weeks and only 42% to 50% of patients naïve from treatment achieve sustained viral response (SVR) [2], [3]. Several specifically-targeted antiviral therapies for HCV (STAT-C) are under development [4]. Telaprevir (also known as VX-950), is a STAT-C that targets the HCV NS3•4A protease and has demonstrated antiviral activity in an HCV replicon assay [5] and in clinical trials [6], [7].

Previously published models of HCV viral dynamics in subjects treated with interferon (IFN), Peg-IFN and RBV have assumed the HCV population within a subject to be relatively homogeneous with respect to sensitivity to these antiviral agents [8],[9],[10],[11]. However, as a consequence of its high replication rate and error-prone polymerase, HCV exists as a quasispecies. In fact, recent data from clinical trials evaluating HCV protease inhibitors have revealed the presence of viral variants with varying levels of sensitivity to these agents [12], [13], [14], [15]. Viral variants have also been detected at levels around 10^−3^ of wild-type NS3•4A HCV (WT) level prior to dosing in treatment-naïve subjects [12], [13]. Upon exposure to protease inhibitors, the composition of the HCV quasispecies was altered, as revealed by sequencing of plasma HCV RNA and isolated viral clones obtained from subjects dosed with telaprevir [14], [15] and boceprevir [13]. These variants have also been reported to exhibit reduced fitness [16], [17] and reduced susceptibility to other protease inhibitors *in vitro* [18].

Models of viral dynamics and emergence of resistance have been developed for viruses like HIV that exhibit a high degree of genetic variability and are capable of establishing chronic infections [19], [20], [21]. In these models, variants were assigned different replicative rates, based either on their infection rate constants, production rate constants, or both. Typically, these models were parameterized using on-treatment HIV RNA levels and CD4+ counts for a small number of resistant variants; however, many of the models did not include sufficient data, in particular the prevalence of variants, to allow estimation of model parameters with good precision. Here, a multi-variant model was developed to represent HCV viral dynamics in subjects dosed with telaprevir monotherapy, to estimate the fitness of variants resistant to telaprevir, and to investigate the importance of replication space dynamics, mutations during treatment, and pre-existing variants on the overall response.

## Methods

### Ethics statement

The study protocol and informed consent form (ICF) were reviewed and approved by an Independent Ethics Committee (IEC) at each of the 3 study centers before initiation of the study. The sites are: Pharma Bio-Research Group BV Medisch Ethische Toetsings Commissie METC Stichting Beoordeling Ethiek Bio-Medisch Onnderzoek P.O. Box 1004 9400 BA Assen, Amsterdam Medical Center Medisch Ethische Toetsings Commissie METC Stichting Beoordeling Ethiek Bio-Medisch Onderzoek P.O. Box 1004 9400 BA ASSEN, The Netherlands Medisch Ethics Toetsings Commissie Meibergdreef 9 P.O. Box 22660 NL 1100 DD Amsterdam, Saarland University Hospital Ärztekammer des Saarlandes Ethikkommission Faktoreistraβe 4 66111 Saarbrücken Germany. Written informed consent was obtained in accordance with the Declaration of Helsinski.

### Subject population and study design

Thirty-four subjects with HCV genotype 1 were enrolled in Study VX04-950-101, a randomized, double-blind, placebo-controlled, 14-day, multi-dose, Phase 1b trial. Subjects received placebo (n = 6) or one of the following dosages of telaprevir administered as a suspension: 450 mg every 8 hours (n = 10), 750 mg every 8 hours (n = 8), or 1250 mg every 12 hours (n = 10). Subjects' baseline characteristics are provided in Supplementary Table S1. Variants were detected using clonal sequencing (details provided in [14]). For the model parameterization described here, data from 26 of the 28 subjects dosed with telaprevir were used. No variants were detected in one subject, and therefore this subject was excluded from further analysis. Estimation results in another subject with 8 variants did not converge to a global optimum---a standard requirement for computationally rigorous estimation; this subject was also excluded.

For each subject, we examined only variants identified by clonal sequencing that were present either at ≥5% of the HCV population at 2 measurement points or ≥10% of the HCV population at 1 time point (5% is the detection limit of the clonal sequencing measurement performed here). The number of variants per subject ranged from 2 to 6; the number of variants for each subject is provided in Supplementary Table S2. These clonal sequencing results identified amino acid differences in HCV NS3•4A that correlated with changes in telaprevir resistance *in vitro*. A larger network representation of quasispecies containing an even greater number of variants could have provided a more complete picture, but was not examined here because no in-subject kinetic data were available to estimate their fitness, and/or no *in vitro* data were available on their resistance to telaprevir.

### A multi-variant viral dynamic model and simulation

The basic evolutionary dynamic among HCV resistant variants in subjects dosed with telaprevir follows Equations 1–5, with variable and parameter descriptions provided in Table 1.
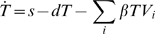
(1)

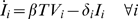
(2)

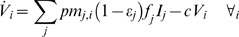
(3)

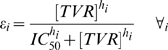
(4)

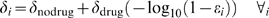
(5)


**Table 1:** Descriptions of model variables and parameters.

Names	Descriptions
(dot above) a variable	time-derivative of a state variable
*T*	healthy target cells, or replication ‘space’
*s*	target cell synthesis rate
*d*	target cell degradation rate constant
*β*	infection rate constant
*V_i_ or V_j_*	plasma virion “i” or “j” with characterized amino-acid substitution(s) and different sensitivities to telaprevir *in vitro*
*I_i_*	*V*_i_-infected cells
*p*	production rate constant of wild-type (WT)
*m_j,i_*	mutation rates from *V*_j_ to *V*_i_
*f_i_*	ratio of production rates of a variant to WT
*c*	plasma virion clearance rate constant
*ε_i_*	production blockage factor of telaprevir to variant *i*
*[TVR]*	effective telaprevir concentration for the observed inhibitions to WT and variants, deduced from the sensitivity curve measured in replicon cells
IC_50,i_	IC_50_ of variant *i* to telaprevir as measured in replicon cells
*h*_i_	Hill coefficient of exponentiation to represent the inhibition curve of variants by telaprevir as measured in replicon cells
*δ_0_*	infected-cell clearance rate constant in subjects dosed with pegIFN and RBV
*δ_1_*	additional infected-cell clearance rate constant in subjects dosed with telaprevir

Variant *V*_i_ represents a virion with characterized amino-acid substitution(s) and *in vitro* defined telaprevir sensitivity. Variant *V*_i_ infects target cells *T* to form variant-*i*-infected cells *I*_i_ at rate *βTV*_i_. It is assumed that each infected cell *I*_i_ is infected by only one variant, and each variant competes for the same target cells *T*. The assumed single-variant infection is consistent with the fact that recombination in HCV appears rarely [22]. Target cells *T* also represent limited replication “space” shared by all variants. Target cells *T* ranges from their baseline level *T*_0_ to their maximum level *T*_max_. Each infected cell *I*_i_ produces a population of variants at production rate *pf_i_*, with fraction *m*_i,j_ mutating to produce variant *j*. The *m*_i,i_ were normalized to follow *m*_i,i_ + ∑_j,j≠ i_ *m*_i,j_ = 1.

Different production rate constants *pf*_i_, but the same infection rate constants (*β*) and clearance rate constants (*c*) are assumed for different variants. The assumption of different production *pf*_i_ is consistent with the function of the NS3•4A protease in cleaving a precursor polyprotein [23], and with variants having been observed with reduced catalytic activity in vitro (data not shown). The production rate ratio *f*_i_ quantifies variant *i* replication disadvantage in the absence of telaprevir. In the presence of telaprevir, the viral production was further reduced by a factor (1- *ε*_i_). The assumed same infection *β* is consistent with lack of interference between HCV protease and HCV envelope proteins. The assumed same plasma clearance *c* is consistent with the data in interferon-based and telaprevir-based treatments [24]. Despite large differences in the antiviral blockages between both treatment groups, similar *c* values were observed. This suggests that *c* is independent of antiviral blockage and therefore, may have the same value among variants. Alternative model formulations with different variants fitness assigned to infection rates or to plasma virion clearance rates produced similar dynamics (Supplementary Figure S3).

Antiviral activities of telaprevir were implemented by assuming a dual role. Telaprevir blocks the production of HCV by inhibiting the activity of the NS3•4A protease with blockage factors *ε*_i_ calculated using Equation 4. The blockage factors for all variants within a subject were calculated using a single effective telaprevir concentration *[TVR]*, with its value estimated from the HCV RNA, variant prevalence dynamics within each subject, and *in vitro* susceptibility of variants to telaprevir. The susceptibility factor *IC_50_*_,i_ and Hill coefficient *h*_i_ were estimated from *in vitro* susceptibility of variant *i* to telaprevir [16], [17] and were provided in Supplementary Table S3. The second role of telaprevir is to enhance infected cell clearance *δ*, a parameter contributing to the second-phase decline. WT *δ*_WT_ values were up to 10-times higher in subjects dosed with telaprevir than in subjects treated with Peg-IFN/RBV [24]; only a 0.2-fold increase in the second-phase decline is explained by increased telaprevir blockage alone (detail calculations are provided in the Supplementary Text S1). On the other hand, in the limit when a subject is not dosed with telaprevir, *δ*_i_ should converge to the clearance without drugs *δ*_nodrug_. These observations were incorporated into the model by assuming that *δ*_i_ increased proportionally to the logarithmic of blockage factor (1-*ε*_i_), given in Equation 5. We also examined alternative models to Equation 5, given by Equations 6 or 7.
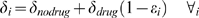
(6)

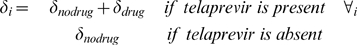
(7)


Prior to dosing, the differential equations were initialized at steady-state. The steady-state initialization is consistent with years of chronic HCV infection. This steady-state solution was used to predict the pre-dosing variant prevalence. During dosing with telaprevir, replication rates of WT and variants were reduced by factors proportional to their sensitivity to telaprevir (blockage factors). Following completion of telaprevir dosing, these blockages were removed. Consequently, the WT and variants present would compete for available replication space with competitive advantages governed by fitness of WT and variants in the absence of any drug.

The majority of the results were reported with replication space *T* described by Equation 1. We also examined another representation of *T* given by Equation 8 [10]. The values of *T*_0_ and *I*_0,i_ were fixed prior to estimation to the steady-state values of *T* and *I*_i_ obtained from models with Equation 1. To obtain a similar rate of *T* increase, the regeneration rate γ were related to parameters in Equation 1 by γ = s/(*T*_0_+∑_i_ *I*_0,i_).
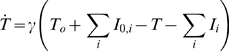
(8)


### Calculation of mutation rates

Previously reported HCV mutation rates range from 1.5×10^−3^ nucleotide changes/site/y [25] to 5×10^−3^ nucleotide changes/site/y [26]. These values were converted to per nucleotide position per replication cycle by assuming an average duration of the HCV replication cycle of 9.5 days calculated as (1/*c*+1/*δ*) with typical values for *c* and *δ* assumed to be the same as those from Peg-IFN/RBV treatments [8], [9]. These calculations resulted in a mutation rate *m* of 1.2×10^−4^ nucleotide changes/site/cycle. The estimations were repeated for different mutation rates of 1.2×10^−2^, 1.2×10^−3^ and 1.2×10^−5^ nucleotide changes/site/cycle.

The mutation rates were computed prior to each estimation by assuming a rate of 1.2×10^−4^ per nucleotide position per replication cycle. The specific mutation rates between two variants were computed by exponentiating the mutation rate for a single mutation by the number of nucleotide mutations between these variants. These rates were genotype specific. For example, to produce NS3•4A protease mutation at position 36 V36M, genotype 1a requires a single nucleotide mutation (from codon GTG to ATG), while genotype 1b requires two mutations (from GTT to ATG).

### Parameter estimation

During-dosing and post-dosing HCV RNA levels and post-dosing variant prevalence data from the clinical study described above (previously published in [6], [14]) were used simultaneously to estimate model parameters. This simultaneous estimation allowed fitness estimation in subjects with HCV RNA levels below the clonal sequencing detection limit (100 IU/mL) at the end of telaprevir dosing but with detectable HCV RNA within a week after completion of dosing. The estimation minimized the maximum likelihood objective function. Parameters estimated for each subject included *c*, *δ_1_*, *p*, *f_i_*, and *[TVR]*. Parameter bounds are provided in Supplementary Table S4. Fitness parameter *f*_i_ was estimated for each variant; the number of assessed variants for each subject varies between 2 to 6 (Supplementary Table S2). The remaining parameters were pre-computed prior to estimation runs, including *m_i,j_*, *β* = 0.05 h^−1^, and *δ*_nodrug_ = 0.12 d^−1^, assuming that the clearance without drugs *δ*_nodrug_ is the same as the clearance on Peg-IFN/RBV treatment. Because we do not have direct measurement of target cells nor productively infected cells, we were able to estimate only the overall viral replication rates, or the basic reproductive ratio *R_0_*_,WT_ =  *pβT*_max_/*(cδ)*. If measurements of infected cells and target cells were available, parameters *p*, *β*, *s*, and *d* may be adjusted to match the measured infected and target cells while maintaining the constraint that *R*_0,WT_ remains constant. For a given R_0,WT_ value, some degree of freedoms exist in choosing *p*, *β*, *s*, and *d*, while clearance parameters *c* and *δ* would be constrained by the decline kinetics. Numerically, this is implemented by fixing parameters *β* and the *s/d* ratio, and normalized HCV RNA levels to the baseline value similar to the implementation in [19]. In the base run, we chose *β* = 0.05 h^−1^ and *T*_max_ =  *s/d = *10, and obtained *R*_0,WT_ from estimates of *p*. These choices of *β* and *T*_max_ prior to estimation did not change the estimates of other parameters estimated from these data, including clearance rates *c*, *δ*, *R*_0,WT_, and fitness *f*_i_. For example, re-estimation with different *β* values, shown in Supplementary Figure S2, resulted in similar fitness and *R*_0,WT_ values. The results also demonstrated an inverse relationship between estimated *p* and assumed *β*, supporting the fact that we were only able to estimate *R_0_*_,WT_, but not *p* nor *β* individually. The breakdown of replication parameters constituting *R*_0,WT_ (*p*, *β* and *T*_max_) may be refined in future studies when direct measurements of target and infected cells become available. Susceptibility factors IC_50,i_ and *h*_i_, were fixed during each estimation. The robustness of the estimates as a function of the dynamics of target cells *T* synthesis rate *s* was also examined by comparing the case when *s* was estimated to the case when *s* was fixed to 1 h^−1^.

### Comparison to relative fitness method

In addition to the modeling approach described in details, we also computed relative fitness (RF). For a variant *i* at two consecutive times *t*_1_ and *t*_2_ with with viral loads *V* ^i^_t1_ and *V* ^i^_t2_, the RF was computed from data from the equation below. If the prevalence was below the detection limit, the value was assumed to be at the limit (5% in this study). Model-derived RF was computed similarly, except that rather than evaluating viral load changes at two consecutive times, the change was evaluated at a specified time *t* using time-derivatives.
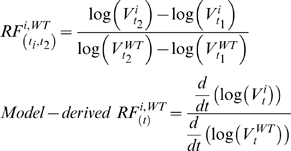



### Numerical implementation

The simulations were implemented by normalizing the plasma virion value with the baseline values obtained after solving the steady-state initial condition. The clearance and replication rates, the balance of which is implicit in the baseline viral load, were estimated directly from HCV RNA decline (during dosing) and rebound (after dosing). The simulation and estimation were implemented using Jacobian Software (R) (RES Group Inc.), using methods described in [27], [28], [29], [30], [31]. Additional information is provided in Supplementary Text S1.

## Results

### Correspondence between data and model-fit

**Figure 1:**
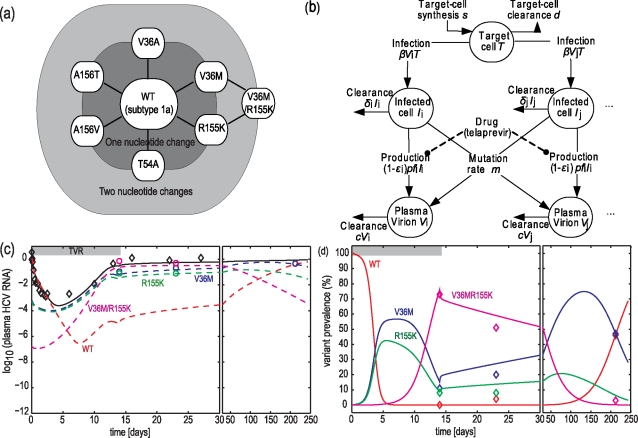
A multi-variant viral dynamic model to quantify response to telaprevir treatment. **Panel** **a**, Superset of HCV genotype 1a variants uncovered in subjects dosed with telaprevir. Each node represents a variant of which the amino acid mutation is printed. Only variants detected in ≥5 subjects are shown. Variants were within 2 nucleotide changes from wild-type HCV. **Panel** **b**, Schematic of the model. **Panels** **c** and **d**, correspondence between data and best-fit model for Subject 1. Diamonds, data; solid line, best-fit model; dashed lines, predicted variant contribution to the overall plasma HCV RNA; circles, HCV RNA levels of variants.

A parameterized multi-variant viral dynamic model was developed to represent the antiviral responses of subjects to telaprevir and to estimate the fitness of variants resistant to telaprevir. Descriptions and schematic of the model is shown in Figure 1. The list of major variants and the nucleotide distances between them are shown in Figure 1a. A schematic of the model is shown in Figure 1b and described by Equations 1–5. Replicative fitness of variants was represented by their different production rate constants *pf*_i_. The basic reproductive ratio of WT HCV *R_0,WT_*, relative fitness *f*_i_, and clearances *c*, *δ* were estimated from time series of both plasma HCV RNA and variant prevalences, with details provided in the Methods section.

The results of the best-fit model corresponded well with observed data. Results in Figure 1c and Figure 1d show an example of a subject who received 450 mg telaprevir q8h for 2 weeks, and whose plasma HCV RNA levels rebounded on dosing. The correspondence between data and model for additional subjects is provided in Supplementary Figure S1. Assuming a pre-dosing steady-state, the best-fit model predicted that HCV with WT NS3•4A protease dominated the HCV quasispecies population as the most fit variant in the absence of any drug, and variant prevalences were near the levels predicted from HCV mutation rates. Upon dosing with telaprevir, WT declined rapidly. Resistant variants also declined initially because of reduced influx of new mutations from WT and, in variants with low-level resistance, because of blockage by telaprevir (the assessment of relative contribution is provided in later section). As the total viral load declined, available replication space was predicted to increase. Such an increase, along with sufficient on-dosing fitness of variants, is necessary for emergence of variants. Had *T* remained constant, none of the variants would have rebounded because each variant would have had a reduced replication flux due to the reduced number of WT available to generate the variant. For the subject shown in Figure 1c and Figure 1d, variants with a single mutation (V36M or R155K) or a double mutation (V36M/R155K) within their NS3•4A protease were responsible for the increase in HCV RNA levels detected initially on Day 6. WT levels were predicted to increase again around Day 8 because of back mutations from variants. When telaprevir dosing was stopped, WT, V36M, and R155K variants out-competed the V36M/R155K variant, and WT eventually regained dominance of the HCV quasispecies population to reach a predicted level of ≥95% of the viral population in 300 days, although V36M persisted for up to 200 days in this subject. The model predicted that immediately after dosing was stopped, V36M initially out-competed WT for available replication space because it was relatively fit and it was 10^4^-times more prevalent. V36M persisted because infected-cell clearance was relatively slow.

### Overall fitness estimates

The estimated fitness obtained from 26 subjects suggests reduced replicative capacity of all telaprevir-resistant variants analyzed compared to WT. Table 2 summarizes estimated production rate ratio (*f*) for all variants, which ranges from 1% to 68% of WT replication. Estimation errors associated with the fitness values were reported as the standard deviation of the estimation error; the errors ranged between 0.03 and 0.12 for variants detected in ≥4 patients, and between 0.4 and 3.26 for variants detected in ≤2 patients. The estimate errors were large especially for variants detected in ≤2 patients that the fitness estimates must be interpreted with cautions. The variants were sorted based on their resistance to telaprevir as measured in replicon cells. The first 7 variants (R155M to A156S) are low-level resistant variants (defined as variants with IC_50_ ≤ the mean estimated effective telaprevir concentration *in vivo* when telaprevir is dosed orally at 750 mg q8h). Among all variants, V36M and R155K single mutant variants with low-level telaprevir resistance have the highest *f* values of 0.68 and 0.66, respectively. Among the high-level resistant variants, the double mutant V36M/R155K had the highest *f* of 0.51.

**Table 2:** Estimates of variants replication rate relative to wild-type HCV (*f*) and corresponding predictions of their pre-dosing prevalence.

Variants^a^	Genotype	Nucleotide	IC_50_	N^b^	Fitness	Precision	Predicted pre-dosing prevalence
		changes from WT	relative to WT^c^		mean _(SD)_ relative production *f* d	median _(range)_ SD of the estimation error^e^	mean _[lower, upper]_ f
R155M	1a	1	5.5 (L)	2	0.01 _(n.a.)_	n.a.	1.2 _[n.a, n.a.]_•10^−4^
T54A	1a	1	6.3 (L)	15	0.55 _(0.24)_	0.10 _[0.03-8.62]_	2.6 _[1.3, 70]_•10^−4^
T54S	1a,1b	1	ND^g^ (L)	2	0.58 _(0.04)_	1.11 _[1.01-1.21]_	2.8 _[n.a., n.a.]_•10^−4^
V36M	1a	1	7.0(L)	12	0.68 _(0.16)_	0.03 _[0.01-2.44]_	3.7 _[2.0, 13]_•10^−4^
R155K	1a	1	7.4(L)	12	0.66 _(0.17)_	0.04 _[0.01-2.79]_	3.4 _[1.8, 12]_•10^−4^
V36A	1a,1b	1	7.4(L)	21	0.49 _(0.21)_	0.04 _[0.02-7.01]_	2.3 _[1.4, 10]_•10^−4^
A156S	1b	1	9.6(L)	2	0.17 _(0.07)_	3.26 _[0.09-6.43]_	1.4 _[1.2, 1.6]_•10^−4^
R155T	1a	1	19.8(H)	4	0.22 _(0.13)_	0.06 _[0.01-0.08]_	1.5 _[n.a., n.a.]_•10^−4^
V36M/R155K	1a	2	≈62(H)	9	0.51 _(0.14)_	0.12 _[0.04-0.66]_	8.8 _[4.0, 40]_•10^−7^
A156T	1a,1b	1	>62(H)	7	0.14 _(0.09)_	0.06 _[0.04-6.57]_	1.4 _[1.2, 1.6]_•10^−4^
A156V	1b	1	>62(H)	5	0.10 _(0.08)_	0.04 _[0.00-5.88]_	1.3 _[1.2, 1.5]_•10^−4^
V36M/T54S	1a	2	ND^h^ (H)	2	0.31 _(0.20)_	0.40 _[0.18-0.61]_	4.1 _[n.a., n.a.]_•10^−7^

a Only variants detected for more than one subject are shown.

b Number of subjects from whose data variant fitness was estimated.

c IC_50_ values were measured in replicon cells; (L) variant with low-level resistance; (H) variant with high-level resistance.

d Mean and standard deviation (SD) computed among subjects.

e The precision of the fitness estimates, standard deviation (SD) of the estimation error in the point estimates of optimal parameter values, was calculated for each subject. The median and range was reported among subjects. N.a. not available (because the optimal value is at lower bound).

f 95% confidence intervals were computed only for variants whose fitness was estimated from more than 5 subjects.

g In the estimation, the IC_50_ of T54S was assumed to be the same as that of T54A.

h In the estimation, the IC_50_ of V36M/T54S was assumed to be the same as that of V36M/R155K.

### Comparison of the fitness estimates from viral dynamic modeling and from the Relative Fitness

**Figure 2:**
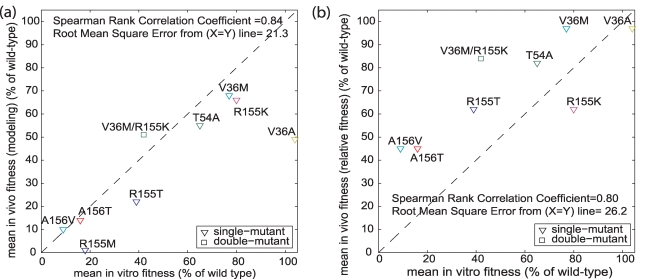
Correspondence between *in vitro* replicative fitness and *in vivo* fitness computed using two alternative methods. **Panel a**, *in vivo* fitness computed using modeling proposed here. **Panel b**, *in vivo* fitness computed using the Relative Fitness method. The *in vitro* fitness was measured in replicon cells and has been reported in [16], [17]. When compared to fitness estimates *in vitro*, *in vivo* fitness estimates from modeling correspond better than the estimates from the Relative Fitness method. Relative Fitness was computed as the ratio of rate of change of viral loads (in log-scale) between a variant and wild-type (see methods).

Previously, we reported fitness estimates based on variants' growth in a subset of subjects [14], using a method similar to the relative fitness (RF) approach [32], [33], normalized to 0–100 scale. In contrast to our earlier RF estimates [14], the fitness estimates herein included data of on-dosing HCV RNA and 3–7 month clonal sequencing, and was expanded to include more subjects (n = 8 for previous estimates, n = 26 for current estimates). The correspondence between these previously reported *in vivo* fitness estimates, the current *in vivo* fitness estimates presented herein, and the *in vitro* fitness estimates reported by others [16], [17] are provided in Figure 2. Figure 2a shows the correspondence of the current *in vivo* estimates to the *in vitro* fitness estimates. With the exception of estimates for variant V36A (which appeared to be an outlier in the *in vitro* estimates), all these fitness estimates were in good agreement. In contrast, Figure 2b shows correspondence of our previous *in vivo* RF estimates [14] with the *in vitro* estimates: the *in vivo* fitness estimates of many of the variants were higher than those of *in vitro* fitness. Thus, compared to the RF method, the fitness estimates presented herein were more consistent with both clinical data [14] and the fitness estimates as measured *in vitro*. For example, current estimates suggest that variant V36M/R155K is less fit than variant R155K, but RF estimates suggested otherwise. The reduced fitness of V36M/R155K is consistent with the data that show increased prevalence at later times (Day 21–23 vs. Day 14; Month 3–7 vs. Day 21–23) of WT, V36M and R155K as compared to the decreased prevalence of V36M/R155K.

An example from a subject (Subject 2, Supplementary Figure S1a and b) demonstrated why fitness estimated from the current model corresponded better with all data than the fitness estimated using the RF method [14]. For this subject, the clonal sequencing data show that although both WT and V36A comprised <5% of the population at Day 14, both were detectable at Day 21, with V36A prevalence relative to that of WT increasing between Days 14 and 21. The current model predicted that at Day 14, when telaprevir dosing was discontinued, V36A HCV RNA levels were about 2-log higher than those of WT. Thus, despite the reduced fitness of V36A over WT, V36A would continue to infect the majority of the target cells T between Days 14 and 21. However, by Day 150, WT dominated the quasispecies because of its higher fitness. If we computed RF of V36A (vs. WT) based only on prevalence data at Day 14 and 21 (*RF*_D14 vs. D21, V36A vs. WT_) and, assuming the same Day 14 prevalence levels of 5%, then *RF*_D14 vs. D21, V36A vs. WT_  = 0.337, a value >0 that misleadingly implies that V36A is more fit than WT. However, this conclusion is inconsistent with the RF calculated between Day 21 and Day 150 (*RF*_D21 vs. D150, V36A vs. WT_) of −0.101, a value <0 which implies that WT is more fit than V36A. On the other hand, the modeling herein estimated *f*_V36A_ *= *0.578, and by using the on-dosing data, correctly accounts for higher V36A levels than WT levels at Day 14, higher V36A levels at Day 21, and reduced levels of V36A at Day 150. Based on the current modeling results, we also can calculate RF values at specific timepoints using a time-derivative of the HCV RNA levels (model-derived *RF*, Table 3). For the V36A variant, the Day 15 model-derived RF was much higher than the RF at Day 100 (−0.671 vs. 0.023), demonstrating dependency of RF values on the specific timing of sample collection. This example demonstrates the advantages of the modeling approach for estimating variants' fitness.

**Table 3:** Fitness estimates using relative fitness and production rate ratio (*f*) in Subject 2.

V36A (vs. WT)	Values
*RF*_D14 vD21_	0.337
*RF*_D21vD150_	−0.101
*f*	0.578
Model-derived *RF_Day15_*	−0.671
Model-derived *RF_Day100_*	−0.023

Relative Fitness (RF) was calculated from data as the ratio (of a variant and wild-type) of the rate of change of viral loads (in log scale). Model-derived RF was calculated similarly, with the rate of change of viral loads from time-derivative of the simulated viral loads at a specified time (see methods).

### Likelihood of variants pre-existing prior to dosing

**Figure 3:**
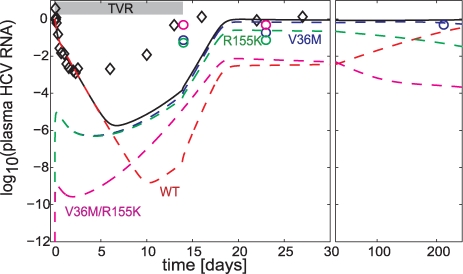
Perturbation analysis of Subject 1, had resistant variants not pre-existed prior to dosing. The simulation was initialized with resistant variants not pre-existed at 0.4 d before dosing; the duration of 0.4 d was chosen as the minimum duration for the plasma HCV RNA of variants to reach steady-state by time = 0. Legends: diamonds, data; lines, models with no variants present at 0.4 day before dosing. Had variants not pre-existed prior to dosing, HCV RNA rebound is expected to occur at later time.

To examine the likelihood of these resistant variants pre-existing before dosing, the time necessary to generate these variants, if they did not pre-exist, was estimated. The best-fit model for Subject 1 above was reinitialized with an HCV population consisting only of WT before dosing started. The results are provided in Figure 3. Had the HCV population consisted only of WT before dosing, the predicted HCV RNA rebound on dosing would be delayed compared to the observed rebound. The poorer (delayed) fit of this modified simulation compared to the one started with a steady-state level of variants before dosing further highlights the likelihood that these variants pre-exist at a steady-state level. Because most subjects in this study have been infected with HCV for years, plenty of time was available for the variants to reach their steady-state levels prior to dosing.

### Sensitivity to different replication space dynamics

**Figure 4:**
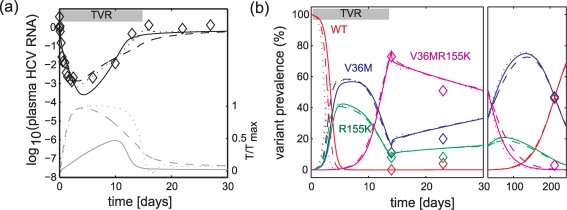
Comparison between data and best-fit models for alternative cases of replication space *T* dynamics applied to Subject 1. **Panel** **a**, comparison for plasma HCV RNA. **Panel** **b**, comparison for variant prevalence composition. Legends: black lines, overall HCV RNA load; grey lines, fraction of available replication space *T/T*_max_; colored lines, contribution of variants to HCV RNA load; solid lines, *T* followed Equation 1 and *s* was optimally estimated at 0.03 h^−1^; dashed lines, *T* followed Equation 1 and *s* was fixed at 1 h^−1^; dotted lines, *T* followed Equation 8 and γ was fixed at 0.05 h^−1^ — a value comparable to *s* = 1 h^−1^ in Equation 1. Alternative representations of similar rate of increase in replication space *T* provide qualitatively similar fits to data.

**Figure 5:**
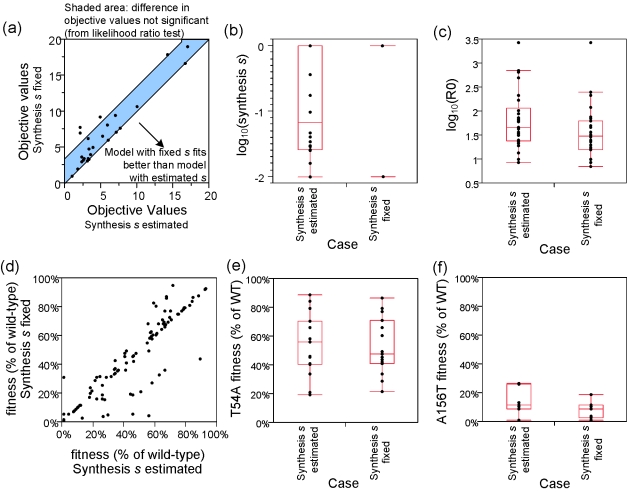
The roles of replication space (*T*) kinetics to model estimates. Two cases of *T* dynamics were examined: first, *T* synthesis rate *s* was estimated (range: [0.01–1] h^−1^) simultaneously with other parameters; second, *s* was fixed to 1 h^−1^. **Panel a**, Maximum likelihood objective function values for both cases. Majority of values are within the objective function differences expected from likelihood ratio for one additional parameter estimated. **Panel b**, Boxplot of synthesis *s* values. **Panel c**, Boxplot of log_10_ of reproductive ratio. Estimates of reproductive ratio is lower when *s* is higher (when s was fixed to its upper bound of 1 h^−1^). **Panel d**, Fitness *f*_i_ for both cases. Similar values suggests robustness to assumed synthesis rate *s* in both cases. **Panels e–f**, Boxplot of fitness *f* values of two representative variants T54A and A156T for both cases.

To understand the contribution of replication space dynamics to the rebound dynamics of resistant variants, we examined three cases: 1) target cells *T* followed Equation 1 with synthesis rate *s* estimated, 2) *T* followed Equation 1 with *s* fixed to its upper bound (1 h^−1^), and 3) *T* followed Equation 8. For these three cases applied to Subject 1, all models corresponded well with observed data (Figure 4), suggesting robustness of the models to these assumptions of *T* dynamics. For *T* dynamics represented by Equation 1, increasing *s* implies faster dynamics for target cells to reach their maximum levels (*T*_max_), resulting in an earlier HCV RNA rebounds. The objective values for the first two cases (*s* estimated and *s* fixed) for all subjects are shown in Figure 5a. The third case was not applied to all subjects because this case has different underlying equations compared to the first two cases --- making a comparison of objective functions difficult. Both cases of *s* produced similar objective values, suggesting robustness of the model fits to the *s* values in the range examined. The estimated *s* values (Figure 5b) had a logarithmic median of 10^−0.88^ h^−1^, a value comparable to the regeneration rate of liver tissues (10^−0.3^–10^−0.6^ h^−1^) [34]. The optimal estimates of *R_0_*_,WT_ varied with *s*, with median (range) log_10_ values of 1.66 (0.93, 3.43) and 1.49 (0.85, 3.43) for estimated *s* and fixed *s* cases respectively, and higher *s* correlated to lower estimates of *R_0_*_,WT_ (Figure 5c). However, the estimates of the production rate ratio *f*_i_ were more robust to the *s* values (Figure 5d–f).

### Sensitivity to different mutation rates

**Figure 6:**
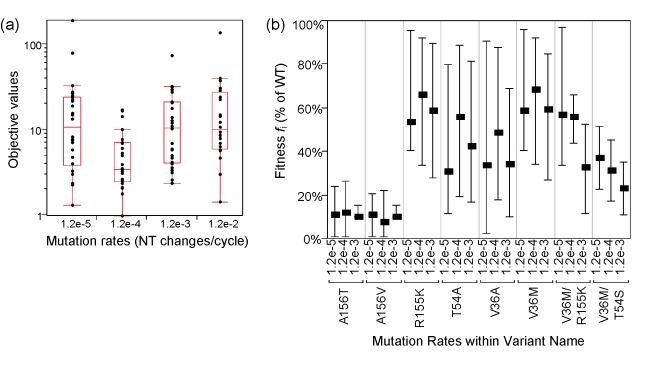
Estimation results for different values of mutation rates *m*. **Panel a**, maximum likelihood objective values for mutation rates *m* of 1.2×10^−5^/cycle, 1.2×10^−4^/cycle, 1.2×10^−3^/cycle, and 1.2×10^−2^/cycle. The objective functions are the lowest with *m* = 1.2×10^−4^/cycle, suggesting models fit data best with this *m* value. **Panel b**, estimated fitness of selected variants at different *m* values. Similar ranking of fitness estimates were obtained with these different values of mutation rates. Similar fitness estimates suggests robustness to the assumed mutation rate *m*. Fitness estimates for m = 1.2×10^−2^/cycle were not reported because of poor model fits.

In the base runs, the mutation rates were assumed to be random with no effect of evolutionary selection, using a value reported from data including evolutionary selection [26]. The inclusion of evolutionary selection may result in mutation rates that underestimated actual rates. Because of this difference in the inclusion of selection to the assumed base mutation rate, we further examined the sensitivity of the estimation results to the assumed rates by repeating the estimations with 10-fold lower, 10-fold and 100-fold higher rates. The results were provided in Figure 6. The objective function values were the lowest for the base case with *m* of 1.2×10^−4^ changes/site/cycle, suggesting that this rate produced the best correspondence between data and model. The ranking of estimated fitness *f*_i_ was qualitatively similar in the three lowest *m* values explored, suggesting that the fitness estimates were robust to the assumption of *m* values in the range of [1.2×10^−5^, 1.2×10^−3^] changes/site/cycle (*f*i for the highest *m* were not reported because of poor model fits). The fitness estimates of the double mutant variants (V36M/R155K, V36M/T54S) were affected the most by *m*; lower *m* produced higher estimates of production rate ratio *f* for these variants. This relationship could be explained by the fact that lower *m* corresponded to lower pre-dosing levels of these double mutants, and to correspond to the levels measured at Day 14, the estimation converged to faster estimates of replication rates (or higher *f* values) of these mutants.

### Rationale for the on-dosing increase in infected-cell clearance *δ*

One feature distinguishing the model proposed here from that previously proposed in HIV [19] is the increase of infected-cell clearance *δ* as a function of on-dosing blockage, given by Equation 5. The higher *δ* was motivated by the observation that WT *δ* values were up to 10-times higher in subjects dosed with telaprevir than in subjects treated with Peg-IFN/RBV [24]. This second-phase decline is much more rapid than the 0.2-fold increase in the decline predicted by increased telaprevir blockage alone, a lower bound value calculated when *T* is held constant (similar differences in the estimates were also obtained when *T* was allowed to vary [24]). On the other hand, in the limit of no telaprevir, *δ*_i_ should converge to *δ*_0_. These two limits constrain alternative relationships between *δ* and on-dosing blockage. For the base case, we chose a model in which *δ*_i_ decreases linearly with log_10_(1-*ε*_i_) as given in Equation 5.

**Figure 7:**
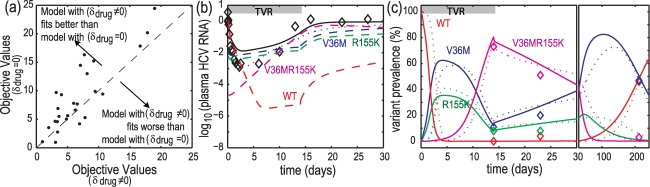
Comparison of cases with (*δ*_drug_≠0) and without (*δ*_drug_ = 0) telaprevir-enhanced infected-cell clearance rate constants. In *δ*_drug_≠0, *δ*_drug_ was estimated from data while *δ*_nodrug_ was fixed at the average value for Peg-IFN/RBV treatment (5.2×10^−3^ h^−1^); in *δ*_drug_ = 0 case, *δ*_nodrug_ was estimated from data while *δ*_drug_ was fixed at zero. **Panel a**, objective values for both cases. The values with *δ*_drug_ = 0 were higher than those with nonzero *δ*_drug_, suggesting better correspondence of data and model fit with *δ*_drug_≠0. The number of parameters estimated in both cases is the same. **Panels b and c**, correspondence between plasma HCV RNA (b) and variant prevalence (c) data and best-fit models for Subject 1. Legends: solid lines, best-fit models with *δ*_drug_ = 0 case; dotted lines, best-fit models with *δ*_drug_≠0 case; dashed lines, variant HCV RNA predicted by best-fit models with *δ*_drug_ = 0 case. Without *δ*_drug_, the best-fit model must trade-off the fitting error on during-dosing second phase decline to match prolonged variants persistence at post-dosing.

To examine the contribution of *δ* enhancement, we compared the correspondence between data and model fits with and without *δ* enhancement. The model with *δ* enhancement (*δ*_drug_ estimated, *δ*_nodrug_ was assumed to be the same as that from Peg-IFN/RBV treatments, and was fixed to the mean value of 0.005 h^−1^) was compared to that without *δ* enhancement (*δ*_drug_ = 0, *δ*_nodrug_ estimated); both models have the same number of estimated parameters. The results are shown in Figure 7. Figure 7a shows the objective values for both models. With the same degree of freedom in both models, the model with *δ* enhancement tends to have lower objective values than that without *δ* enhancement, suggesting better correspondence of the *δ* enhancement model to the data. Figure 7b and c show the example of the correspondence of model without enhancement applied to Subject 1. The model without enhancement missed the much more rapid on-dosing HCV RNA declines while maintaining the fit to variant prevalence data. These observations suggest that δ enhancement affects both WT and variants.

**Figure 8:**
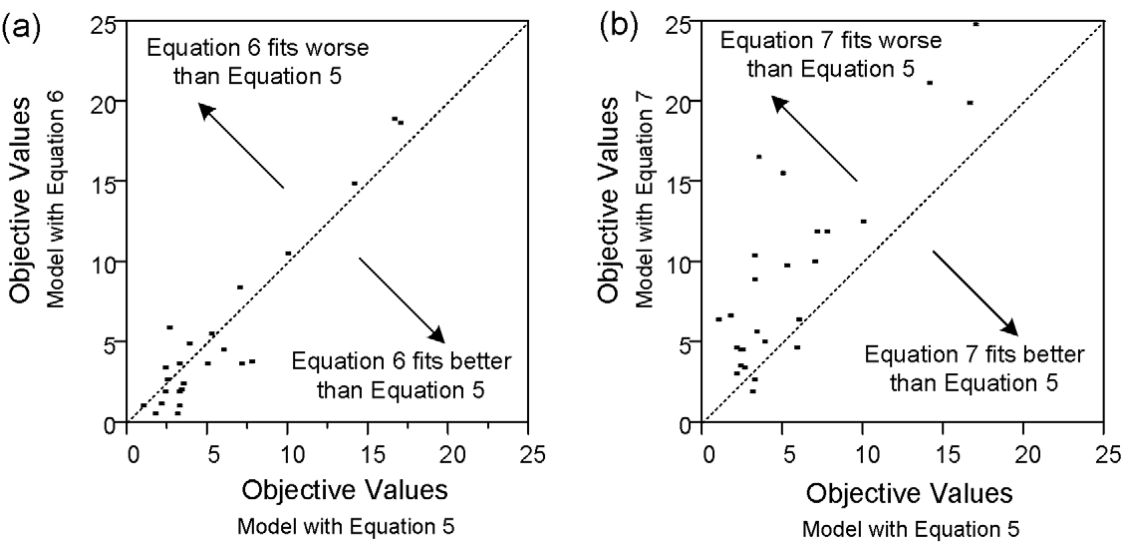
Objective values for alternative equations representing enhanced infected-cell clearance rates *δ* (Equations 5, 6, and 7). Each of the models have the same number of parameters estimated for each subject. Models with *δ*_i_ that depend on blockage *ε* (Equations 5 and 6) have similar quality of fits. However, the model without dependency to *ε* (Equation 7) has inferior quality of fits.

To represent the *δ* enhancement in WT and variants, while satisfying the two limits of the observed second slope described above, we also examined alternative equations to Equation 5. In particular, we examined models with *δ*_i_ as a linear function of (1-*ε*_i_) (Equation 6) and models with *δ*_i_ as a step-function to the presence of telaprevir (Equation 7). Because the number of parameters estimated in each patient are the same, one may compare the objective functions directly to represent the goodness of fit. The results are provided in Figure 8. Compared to the base model with Equation 5, the model including Equation 6 produced similar quality of fit. However, the model with Equation 7 produced inferior fits. These suggest that the increase in the second slope is blockage-dependent.

### Relative contribution of factors leading to an initial decline of variants levels on dosing

Upon dosing with telaprevir, variants' RNA levels were predicted to decline initially because of two factors: blockage of replication by telaprevir and reduced influx mutations from WT due to rapid WT clearance. To examine the relative contributions of these two factors, the model components were examined at the beginning of dosing. At this timepoint, the reduction of variant *i*'s replication flux by telaprevir blockage can be approximated as -*ε*_i_ *f*_i_ *I*_i_, and the reduction of influx mutation by WT clearance can be approximated as *m*_WT,i_ *f*_WT_ *I*_WT_. Thus, assuming that prior to dosing *V*_i_/*V*_WT_  =  *m*_WT,i_/(1-*f*_i_), the ratio of reduced replication of variant *i* by blockage to that by mutation can be approximated by ε_i_ *f*_i_/(1-*f*_i_). If we assumed an effective telaprevir concentration *[TVR]* of 4 µM (the average *[TVR]* in the cohorts studied here) and calculated *ε* from Equation 5, then for the V36M variant with intermediate susceptibility (IC_50_ = 4.73 µM, Hill coefficient *h* = 3.5, *f*_V36M_ = 0.68), this ratio was 0.97. For the A156T variant with high resistance and low fitness (IC_50_  = 10^3^ µM, *h* = 1, f_A156T_ = 0.1), the ratio was 4×10^−4^. For the V36M/R155K variant with high resistance and high fitness (IC_50_ = 142 µM, *h* = 3.5, f_V36M/R155K_ = 0.5), this ratio is 4×10^−6^. The fact that these ratios are all <1 suggests that the reduction in influx mutations from WT, rather than the increased telaprevir blockage, dominated the initial reduction of the variants' replication rates.

## Discussion

The HCV viral dynamics in subjects dosed with telaprevir were represented by a multi-variant model that included the heterogeneity of variants' fitness, and resistant profiles in the HCV quasispecies. During telaprevir dosing, the overall viral load initially declined as WT was inhibited and replication space available to variants increased, allowing pre-existing variants with sufficient on-dosing fitness to emerge. Unlike during HIV infection, where replication space can be quantified by measuring healthy CD4+ cells [35] replication space in HCV-infected subjects cannot be measured directly. However, the concept of limited replication space is important in HCV infection because HCV RNA levels reach a steady-state value in chronically infected subjects, indicating limited resources for viral replication. Biologically, the replication space in HCV may be limited by the number of healthy hepatocytes, or by other factors necessary for viral replication within these cells (e.g., factors for RNA and/or protein synthesis, or for inhibition of the double-stranded RNA induced signaling pathway [36]).

The increase in replication space and the on-dosing fitness of variants were the primary determinants of HCV RNA rebound during telaprevir dosing, with negligible contribution from mutations during treatment. The finding of variants prior to dosing [12], [13] and within a week on-treatment in other studies [15] suggested that the variants contributing to virologic rebound in the present study were likely to pre-exist. The pre-existence of variants is supported by the modeling results; had they not pre-existed, calculations based on the HCV mutation rate, replication rate, and HCV RNA level at baseline indicated that rebound would have been delayed. A complementary computational analysis of HIV viral dynamics has also identified the likelihood of some HIV variants pre-existing [37]. The mechanism of viral rebound (by resistant variants when WT is cleared) is also consistent with predicted behavior in HIV drug resistance models [38], [39]. Moreover, rapid virologic rebound in HIV-infected subjects treated with nonnucleoside reverse transcriptase inhibitors [40], [41] has also been attributed to the pre-existence of variants [42]. A caveat here is that the risk of other (novel) resistant variants being generated on-treatment is higher if HCV is not cleared rapidly. Higher HCV RNA levels translate to more replication cycles and thus, larger risk of developing new variants. The higher risk further highlights the importance of treating HCV-infected subjects with potent regimens.

Based on data observed during the emergence of telaprevir resistant variants, the model described herein estimates a replication space synthesis rate *s* in subjects chronically infected with HCV that is higher than the *s* obtained for subjects chronically infected with HIV [19], and estimated reproductive ratio R0 values that were both higher and more variable compared to those for HIV. The high *s* is consistent with the high regenerative rates of hepatocytes [34]. Additionally, this high *s* may reflect the additional influx of healthy cells as a result of HCV RNA elimination in infected-cells (cured cells), consistent with the observed faster second-phase decline in subjects with HCV dosed with telaprevir as compared to the decline in subjects treated with IFN/RBV [24]. The R0 values for HCV estimated here were about 2-fold higher (median: 45.7; range: 8.51 to 2692) than the values obtained from acute HIV infections (Number of patient N = 5, median: 19.3, range: 7.4 to 34) [43], and about 11-fold higher than the values after interruption of antiretroviral therapy [43]. Because HCV infected-cell clearance rates---a component of R0---may be affected by innate immunity, it is plausible that the higher variability of R0 for HCV may be related to the HCV interference of the innate immunity [44], [45].

All variants resistant to telaprevir estimated here have reduced replicative fitness *in vivo* compared to WT in the absence of telaprevir. The phenotypic and structural bases for the resistance and reduced fitness of these variants have been discussed elsewhere [16], [17]. The reduced fitness is consistent with 98% of HCV-infected subjects having quasispecies dominated by WT prior to dosing [46] and with the finding that after telaprevir dosing was stopped, WT regained its dominance in the quasispecies [14], [15]. Resistant HIV variants also have reduced fitness [47], [48], [49]. However, in contrast to treatments in HIV, treatments in HCV may result in a sustained viral response (clearance of virus). Variants resistant to telaprevir remain sensitive to Peg-IFN/RBV *in vitro* [16], [17], and their reduced fitness may reduce the “load” for Peg-IFN/RBV treatment within a subject, increasing the percentage of HCV-infected subjects responding to Peg-IFN/RBV treatment. This suggests that a shorter treatment duration and/or a higher percentage of subjects reaching SVR may potentially be possible using triple therapy with telaprevir, Peg-IFN and RBV than with Peg-IFN and RBV alone.

Models with on-dosing increase of infected cell clearance provided better fits of the data. Estimates of second slopes of HCV RNA decline during the first three days of dosing attributable to WT HCV dynamics revealed 10-fold increased infected-cell clearance compared to treatment with interferon and ribavirin [24]. Here, we also found that, when compared to models with enhanced infected-cell clearance, models lacking the enhanced clearance are inferior in estimating data. Moreover, we found that models that includes a blockage-dependent second slope fit the data better than models with a switch-like second slope. Biologically, we hypothesized that the phenomenon may be related to the interference of HCV in the innate immune response [44], [45]. Elimination of WT HCV by telaprevir may allow previously WT-dominant infected cells to restore their normal innate immune response while variant levels are low, resulting in a more rapid second-phase decline.

The HCV RNA response in subjects dosed with telaprevir monotherapy and the estimate of variants' fitness have been quantified using a multi-variant viral dynamic model. Here we showed how diversity in viral quasispecies should be accounted for in a model of antiviral response to specifically-targeted antiviral compounds.

## Supporting Information

Text S1 Supplementary text
(0.06 MB DOC)

Table S1 Subjects characteristics
(0.06 MB DOC)

Table S2 Type of variants with fitness estimates for each subject. Four variants were observed only in one subject and are not included. Nsubjects, the number of subjects in which the variant was observed; Nvariants, the number of variants observed within a subject.
(0.12 MB DOC)

Table S3 Variants susceptibility to telaprevir as measured in replicon cells
(0.05 MB DOC)

Table S4 Bounds on the estimated parameters
(0.05 MB DOC)

Figure S1 Correspondence between data and model estimation in three additional subjects. a, Subject 2 HCV RNA levels; b, Subject 2 variant prevalence; c, Subject 3 HCV RNA levels; d, Subject 3 variant prevalence level; e, Subject 4 HCV RNA levels; f, Subject 4 prevalence. Diamonds, data; solid line, best-fit model; dashed lines, predicted variant contribution to the overall plasma HCV RNA; circles, HCV RNA levels of variants (limited to variants with prevalence >5%).
(1.80 MB EPS)

Figure S2 Sensitivity of estimation results to assumption of β values applied to data from Subject 1. Panel A, Maximum likelihood objective values. The objective values were similar despite large variations of fixed pre-estimated β. Panel B, estimated production rate constant p for a given β value. Production rate p is related to 1/β. Panel C, estimated fitness of V36M. Panel D, estimated fitness of R155K. Estimated fitness converged to similar values despite large variations of assumed β values. The estimation was repeated 2000 times for this subject with different β values (with β/β_0_ = 10^βrandom^; β_random_ as a random variable with mean = 0, std = 1). Initial seed and bounds for p were adjusted to maintain constant (pβTmax/(cδ)) values (p/p_0_ = 10^(-βrandom)^; p_0_ as the estimated p when β = β_0_). The same estimates of reproductive ratio R0 and fitness f_i_ were obtained despite extreme ranges of β.
(2.09 MB EPS)

Figure S3 Sensitivity to alternative models with different representation of variant fitness: Baseline: where fitness is represented by different production rates f_i_ p; Case 1, where fitness is represented by different infection rate f_i_ β; Case 2, where fitness is represented by different plasma clearance rate c/f_i_. These alternative models maintain the same variant reproductive ratio R_0,i_ and resulted in similar viral dynamics.
(0.09 MB TIF)
