# Supplementary material for: A Multi-Variant, Viral Dynamic Model of Genotype 1 HCV to Assess the in vivo Evolution of Protease-Inhibitor Resistant Variants
Source: PLoS Comput Biol. 2010 Apr 15;6(4):e1000745. doi: 10.1371/journal.pcbi.1000745 (PMC2855330; doi:10.1371/journal.pcbi.1000745)
Supplement: Text S1 — Supplementary text (0.06 MB DOC) [file pcbi.1000745.s001.doc]

## SUPPLEMENTARY MATERIALS

### Normalization to baseline HCV RNA and choices of *β* and *T*_max_ values

The model in Equations 1-3 were implemented numerically as the normalized form to baseline HCV RNA level (*V*_0_), given as:

$$\begin{matrix} \mathop{\frac{T}{V_{0}}}\limits^{\bullet} = & \frac{s}{V_{0}}-d\frac{T}{V_{0}}-\sum_{i} \beta V_{0}\frac{T}{V_{0}}\frac{V_{i}}{V_{0}} & & (S1) \\ \frac{\mathop{I_{i}}\limits^{\bullet} }{V_{0}}= & \beta V_{0}\frac{T}{V_{0}}\frac{V_{i}}{V_{0}}-\delta_{i}\frac{I_{i}}{V_{0}} & \forall i & (S2) \\ \frac{\mathop{V_{i}}\limits^{\bullet} }{V_{0}}= & \sum_{j} pm_{j,i}(1-\varepsilon_{j})f_{j}\frac{I_{j}}{V_{0}}-c\frac{V_{i}}{V_{0}} & \forall i & (S3) \\ \mathop{t}\limits^{\bullet} = & s'-dt-\sum_{i} \beta'tv_{i} & & (S4) \\ \mathop{i}\limits^{\bullet} _{i}= & \beta'tv_{i}-\delta_{i}i_{i} & \forall i & (S5) \\ \mathop{v}\limits^{\bullet} _{i}= & \sum_{j} pm_{j,i}(1-\varepsilon_{j})f_{j}i_{j}-cv_{i} & \forall i & (S6) \end{matrix}$$

Where $t=\frac{T}{V_{0}};i_{i}=\frac{I_{i}}{V_{0}};v_{i}=\frac{V_{i}}{V_{0}};s'=\frac{s}{V_{0}};\beta'=\frac{\beta}{V_{0}}$. The *V*_0_-normalized model (Equations S4-S6) demonstrated that parameters *s, β* are affected by *V*_0_, but not parameters *d*, δ_i_, *p*, *f*_i_, *c*_i_.

**Sensitivities to alternative assumptions of fitness represented by infection rates or by plasma clearance rates**

In the baseline simulation, different variant fitness was represented by different production rates. The sensitivities of this assumption were examined by examining alternative models that maintain the same reproductive ratio of variants R_0,i_:

1. with fitness represented by different infection rates (assuming the same production rates for all variants). Infection rate of variant *i* *β*_i_ was represented as *β*_i_ = *f*_i_ *β*
2. with fitness represented by different plasma clearance rates (assuming the same production rates for all variants). Clearance rates of variant *i* *c*_i_ was represented as *c*_i_ = *c*/*f*_i_.

The simulation results for a subject are shown in Supplementary Figure S3. The same dynamics were obtained for the baseline and both cases described above, suggesting that alternative models with the same variants reproductive ratio R_0,i_ resulted in the same dynamics. Therefore, estimations with these alternative models are also expected to produce the same results.

### Expected increase in the second-phase decline by increased telaprevir blockage

Assuming a constant replication space *T*, the second phase decline (*λ*_2_) is related to the clearance and blockage parameters according to the following equation: [8,10].

$$\lambda_{2}=0.5\left\{(c+\delta)-[(c-\delta)^{2}+4(1-\varepsilon)c\delta]^{0.5}\right\}$$

If two drugs exist with different blockage factors *ε*_A_ and *ε*_B_ but the same clearances *c* and *δ*, then the expected difference in *λ*_2_ is given by the following equation:

$$\lambda_{2}^{A}-\lambda_{2}^{B}=[(c-\delta)^{2}+4(1-\varepsilon_{B})c\delta]^{0.5}-[(c-\delta)^{2}+4(1-\varepsilon_{A})c\delta]^{0.5}$$

Assuming typical clearance rate constants *c* and *δ* for Peg-IFN and RBV (*c*=10 d^-1^, *δ*=0.2 d^-1^, (1-*ε*)=10^‑0.7^, [8,11]), an increase in blockage from (1- *ε*)= 10^-0.7^ (estimated blockage for WT with Peg-IFN/RBV therapy) to (1-*ε*)=10^‑2.12^ (estimated blockage for WT with TVR monotherapy) would increase the second phase decline by only 0.04 d^-1^, or 0.2-fold. Because the observed second-phase decline in subjects dosed with telaprevir was 10-fold [24], mechanisms other than increased blockage are needed to explain the observed steeper decline.

**Maximum likelihood objective function definition:**

The estimation problem solved an optimization problem with the maximum likelihood estimator [27] implemented within Jacobian Software (Numerica Technology, LLC.) defined below:

Parameter *p* is the set of time-invariant parameters to be estimated, *N* is the total number of measurements of all variables, *N_v_* is the number of measured variables (*N_v_*=2, HCV RNA and variant prevalence), *N_m,j_* is the number of measurements of variable j, $\hat{z}_{jk}$ is the j-th observation of variable k and *z*_jk_(p) is the corresponding value computed from the model, σ_jk_ is the standard deviation of measurement $\hat{z}_{jk}$. In the analysis, the variables estimated were the log_10_ of plasma HCV RNA and variant prevalence (normalized to the scale of 0-10 to allow comparable weighting to the log_10_ of plasma HCV RNA).

**Dynamic optimization setup**

The dynamic estimation problem utilized the control vector parameterization approach [28], where the dynamic optimization was solved as a two-step process consisting of integration and optimization steps. The integration was performed by using the staggered corrector sensitivity analysis method [29]. The optimization was solved using nonlinear programming/NLP approach; implemented using a Successive Quadratic Program method of Large-scale Broyden-Fletcher-Goldfarb-Shanno (LBFGS) [30]. The dynamic simulation was implemented with integration relative and absolute tolerances of 10^-6^. The estimation was implemented using multi-start with random initial seeds, repeated until best local optimum converged to a global optimum.

**Determination of global optimality**

The optimization step in the control vector parameterization was implemented as a nonlinear programming approach and will converge to local optimal solutions. Unfortunately, a rigorously global, nonlinear dynamic optimization remains difficult to implement. Here, we choose to perform multi-start from random initial seeds. For each subject, estimation runs from 300 random initial seeds were performed. We required that the lowest local optimal objective functions (within 10^-4^ relative and absolute tolerance) were obtained for at least 5 times. Furthermore, for these runs with lowest objective values, the optimal parameter values were compared to determine if they converged to similar values. Because of the different objective value sensitivity of these parameters, the convergence criterion for the optimal parameter values were determined by their absolute differences in values, normalized by the sensitivity of the objective function to the parameters at the optimal solution. The multi-start optimization does not guarantee rigorous global optimality; however, the final optimum solution is likely to be the global optimum because of sufficient sampling of parameter space and the good correspondence with experimental data. Details on the normalization have been published previously [31].
