# Supplementary material for: A Multi-Variant, Viral Dynamic Model of Genotype 1 HCV to Assess the in vivo Evolution of Protease-Inhibitor Resistant Variants
Source: PLoS Comput Biol. 2010 Apr 15;6(4):e1000745. doi: 10.1371/journal.pcbi.1000745 (PMC2855330; doi:10.1371/journal.pcbi.1000745)
Supplement: Table S1 — Subjects characteristics (0.06 MB DOC) [file pcbi.1000745.s002.doc]

Supplementary Table S1 Subjects characteristics^a^

|  | **Placebo**  **(n=6)** | **Telaprevir** | | |
| --- | --- | --- | --- | --- |
|  |  | **450 mg q8h**  **(n=10)** | **750 mg q8h**  **(n=8)** | **1250 mg q12h**  **(n=10)** |
| **Sex, n (%)**  **male**  **female** | **3 (50.0)**  **3 (50.0)** | **8 (80.0)**  **2 (20.0)** | **3 (37.5)**  **5 (62.5)** | **8 (80.0)**  **2 (20.0)** |
| **Race, n (%)**  **white** | **6 (100.0)** | **10 (100.0)** | **8 (100.0)** | **10 (100.0)** |
| **Age, y**  **median**  **range** | **54**  **31-64** | **47**  **33-64** | **52**  **46-64** | **43.5**  **(25-62)** |
| **BMI, kg/m2**  **median**  **range** | **24.8**  **21.0-29.0** | **25.8**  **22.6-28.4** | **27.0**  **21.1-29.4** | **22.2**  **21.2-24.3** |
| **baseline HCV RNA, log_10_ IU/mL, mean (SD)** | **6.28 (0.47)** | **6.54 (0.50)** | **6.18 (0.47)** | **6.46 (0.41)** |
| **Prior HCV treatment, n (%)** | **4 (66.7)** | **9 (90.0)** | **7 (87.5)** | **7 (70.0)** |
| **Genotypes**  **1(others)**  **1a**  **1b** | **1 (16.7)**  **2 (33.3)**  **3 (50.0)** | **-**  **3 (30.0)**  **7 (70.0)** | **2 (25.0)**  **1 (12.5)**  **5 (62.5)** | **1 (10.0)**  **5 (50.0)**  **4 (40.0)** |

^a^ All subjects were HIV negative

q8h: dosing every 8 hours

q12h: dosing every 12 hours
