# Supplementary material for: A Multi-Variant, Viral Dynamic Model of Genotype 1 HCV to Assess the in vivo Evolution of Protease-Inhibitor Resistant Variants
Source: PLoS Comput Biol. 2010 Apr 15;6(4):e1000745. doi: 10.1371/journal.pcbi.1000745 (PMC2855330; doi:10.1371/journal.pcbi.1000745)
Supplement: Table S2 — Type of variants with fitness estimates for each subject. Four variants were observed only in one subject and are not included. Nsubjects, the number of subjects in which the variant was observed; Nvariants, the number of variants observed within a subject. (0.12 MB DOC) [file pcbi.1000745.s003.doc]

Supplementary Table S2 Type of variants with fitness estimates for each subject. Four variants were observed only in one subject and are not included. Nsubjects, the number of subjects in which the variant was observed; Nvariants, the number of variants observed within a subject.

|  | Variants Name | | | | | | | | | | | | | |
| --- | --- | --- | --- | --- | --- | --- | --- | --- | --- | --- | --- | --- | --- | --- |
| Randomized Subject ID | Wild Type | A156S | A156T | A156V | R155K | R155M | R155T | T54A | T54S | V36A | V36M | V36M/R155K | V36M/T54S | Nvariants |
| 1 | 1 | 0 | 0 | 0 | 1 | 0 | 0 | 0 | 0 | 0 | 1 | 1 | 0 | 4 |
| 2 | 1 | 0 | 0 | 0 | 1 | 0 | 1 | 0 | 0 | 1 | 1 | 1 | 0 | 6 |
| 3 | 1 | 0 | 0 | 0 | 0 | 0 | 0 | 1 | 0 | 1 | 0 | 0 | 0 | 4 |
| 4 | 1 | 0 | 1 | 1 | 0 | 0 | 0 | 1 | 0 | 1 | 0 | 0 | 0 | 6 |
| 5 | 1 | 1 | 0 | 0 | 0 | 0 | 0 | 1 | 0 | 1 | 0 | 0 | 0 | 4 |
| 6 | 1 | 0 | 1 | 0 | 0 | 0 | 0 | 1 | 0 | 1 | 0 | 0 | 0 | 4 |
| 7 | 1 | 0 | 0 | 0 | 1 | 0 | 1 | 0 | 0 | 1 | 1 | 0 | 0 | 6 |
| 8 | 1 | 1 | 1 | 1 | 0 | 0 | 0 | 1 | 0 | 1 | 0 | 0 | 0 | 7 |
| 9 | 1 | 0 | 0 | 0 | 1 | 0 | 0 | 0 | 0 | 1 | 1 | 1 | 0 | 5 |
| 10 | 1 | 0 | 0 | 0 | 1 | 0 | 0 | 0 | 1 | 1 | 1 | 0 | 1 | 6 |
| 11 | 1 | 0 | 0 | 0 | 1 | 0 | 0 | 0 | 0 | 0 | 1 | 1 | 1 | 5 |
| 12 | 1 | 0 | 0 | 0 | 0 | 0 | 0 | 1 | 0 | 1 | 0 | 0 | 0 | 3 |
| 13 | 1 | 0 | 0 | 0 | 0 | 0 | 0 | 1 | 0 | 1 | 0 | 0 | 0 | 3 |
| 14 | 1 | 0 | 0 | 0 | 0 | 0 | 0 | 1 | 0 | 1 | 0 | 0 | 0 | 3 |
| 15 | 1 | 0 | 0 | 0 | 0 | 0 | 0 | 1 | 0 | 1 | 0 | 0 | 0 | 3 |
| 16 | 1 | 0 | 1 | 0 | 1 | 0 | 0 | 0 | 0 | 1 | 1 | 1 | 0 | 6 |
| 17 | 1 | 0 | 1 | 1 | 0 | 0 | 0 | 1 | 0 | 1 | 0 | 0 | 0 | 5 |
| 18 | 1 | 0 | 1 | 0 | 0 | 0 | 0 | 0 | 1 | 1 | 0 | 0 | 0 | 4 |
| 19 | 1 | 0 | 0 | 0 | 0 | 0 | 0 | 1 | 0 | 1 | 0 | 0 | 0 | 3 |
| 20 | 1 | 0 | 0 | 0 | 1 | 0 | 0 | 1 | 0 | 1 | 1 | 0 | 0 | 5 |
| 21 | 1 | 0 | 0 | 0 | 1 | 1 | 1 | 0 | 0 | 0 | 1 | 1 | 0 | 6 |
| 22 | 1 | 0 | 0 | 0 | 1 | 0 | 0 | 1 | 0 | 1 | 1 | 1 | 0 | 6 |
| 23 | 1 | 0 | 0 | 0 | 1 | 1 | 1 | 0 | 0 | 0 | 1 | 1 | 0 | 6 |
| 24 | 1 | 0 | 0 | 0 | 1 | 0 | 0 | 0 | 0 | 1 | 1 | 1 | 0 | 5 |
| 25 | 1 | 0 | 1 | 1 | 0 | 0 | 0 | 1 | 0 | 1 | 0 | 0 | 0 | 5 |
| 26 | 1 | 0 | 0 | 1 | 0 | 0 | 0 | 1 | 0 | 0 | 0 | 0 | 0 | 3 |
| Nsubjects |  | 2 | 7 | 5 | 12 | 2 | 4 | 15 | 2 | 21 | 12 | 9 | 2 |  |
