# Supplementary material for: A Multi-Variant, Viral Dynamic Model of Genotype 1 HCV to Assess the in vivo Evolution of Protease-Inhibitor Resistant Variants
Source: PLoS Comput Biol. 2010 Apr 15;6(4):e1000745. doi: 10.1371/journal.pcbi.1000745 (PMC2855330; doi:10.1371/journal.pcbi.1000745)
Supplement: Table S3 — Variants susceptibility to telaprevir as measured in replicon cells (0.05 MB DOC) [file pcbi.1000745.s004.doc]

Supplementary Table S3 Variants susceptibility to telaprevir as measured in replicon cells.

| **Variants** | **IC50^a^ (μM)** | **hill power *h*^a^** | **Note** |
| --- | --- | --- | --- |
| WT | 1.06 | 1.66 |  |
| R155M | 3.56 | 3.54 |  |
| T54A | 4.26 | 3.54 |  |
| T54S | 4.26 | 3.54 | assumed the same IC50 and *h* as T54A |
| V36M | 4.73 | 3.46 |  |
| R155K | 4.77 | 3.54 |  |
| V36A | 4.99 | 3.46 |  |
| A156S | 6.47 | 1.00 | assumed similar *h* to A156V/T |
| R155T | 12.75 | 3.54 | assumed similar *h* to R155K |
| V36M/R155K | 142.51 | 3.54 | assumed similar *h* to R155K |
| A156V | 1000.00 | 1.00 | beyond detection limit in replicon, assumed no blockage |
| A156T | 1000.00 | 1.00 | beyond detection limit in replicon, assumed no blockage |

^a^The IC50 and *h* values reported here were obtained from [5,16,17].
