# Supplementary material for: A Multi-Variant, Viral Dynamic Model of Genotype 1 HCV to Assess the in vivo Evolution of Protease-Inhibitor Resistant Variants
Source: PLoS Comput Biol. 2010 Apr 15;6(4):e1000745. doi: 10.1371/journal.pcbi.1000745 (PMC2855330; doi:10.1371/journal.pcbi.1000745)
Supplement: Table S4 — Bounds on the estimated parameters (0.05 MB DOC) [file pcbi.1000745.s005.doc]

Supplementary Table S4 Bounds on the estimated parameters

| **Parameter Name** | **Description** | **Unit** | **Lower Bound** | **Upper Bound** |
| --- | --- | --- | --- | --- |
| *p* | production rate constant | d^-1^ | 0.24 | 120 |
| *c* | plasma viral clearance rate constant | d^-1^ | 0.12 | 24 |
| *δ*_drug_ | infected cell clearance rate constant | d^-1^ | 0.024 | 2.4 |
| *[TVR]* | effective telaprevir concentration | μM | 1.5 | 20 |
| *s* | target cell synthesis rate | d^-1^ | 0.24 | 24 |
| *f_i_* | variant production rate ratio to WT in the absence of drug | unitless | 0.01 | 0.99 |
