# Supplementary figures and images for: A Multi-Variant, Viral Dynamic Model of Genotype 1 HCV to Assess the in vivo Evolution of Protease-Inhibitor Resistant Variants
Source: PLoS Comput Biol. 2010 Apr 15;6(4):e1000745. doi: 10.1371/journal.pcbi.1000745 (PMC2855330; doi:10.1371/journal.pcbi.1000745)

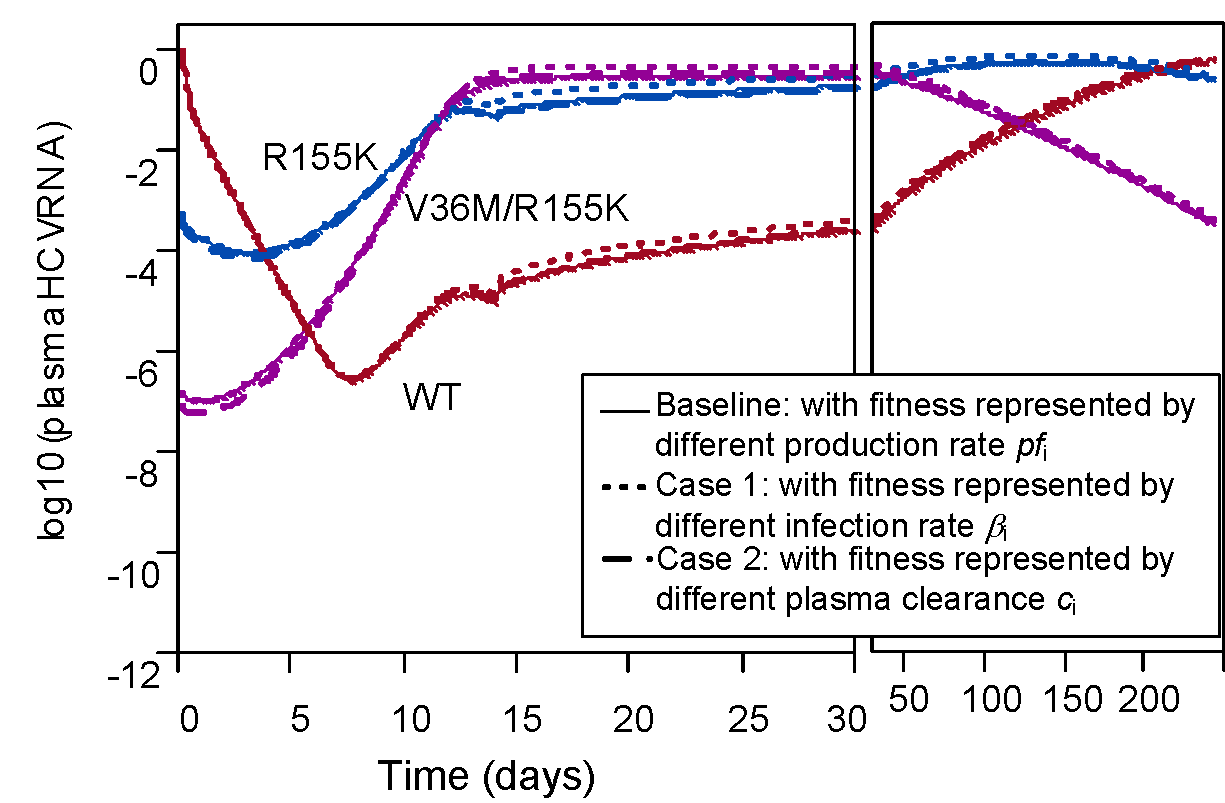

Supplement: Figure S3 — Sensitivity to alternative models with different representation of variant fitness: Baseline: where fitness is represented by different production rates fi p; Case 1, where fitness is represented by different infection rate fi β; Case 2, where fitness is represented by different plasma clearance rate c/fi. These alternative models maintain the same variant reproductive ratio R0,i and resulted in similar viral dynamics. (0.09 MB TIF) [file pcbi.1000745.s008.tif]
